# Androgen Plays a Carcinogenic Role in EOC via the PI3K/AKT Signaling Pathway in an AR-Dependent Manner

**DOI:** 10.7150/jca.51099

**Published:** 2021-01-21

**Authors:** Yanfang Li, Sha Li, Yizhou Zhang, Shuhong Shi, Shan Qin, Chang Wang, Juan Du, Jingle Ma, Huan Chen, Huixian Cui

**Affiliations:** 1 Department of Anatomy, Hebei Medical University, Shijiazhuang, China; 2 Department of Obstetrics and Gynecology, The Second Hospital of Hebei Medical University, Shijiazhuang, China; 3 Neuroscience Research Center, Hebei Medical University, Shijiazhuang, China.; 4 Hebei Key Laboratory of Neurodegenerative Disease Mechanism, Shijiazhuang, China.

**Keywords:** ovarian carcinoma, androgen receptor (AR), tissue microarray (TMA), proliferation promotion, PI3K/AKT signalling pathway

## Abstract

**Background:** Epithelial ovarian cancer (EOC) is one of the most common gynecological cancers with the highest mortality rate. Studies indicate that androgens contribute to initiation or progression of EOC through poorly understood mechanisms, however, in the phase II clinical studies of antiandrogen therapy for EOC, neither flutamide nor bicalutamide showed good antitumor effects. Based on the contradictions, the purpose of this study was to explore the role of androgen receptor (AR) in the androgen pathogenesis of EOC and the possible mechanism, and further to find an indicator to screen the anti-androgen therapy sensitive cases.

**Methods:** In this study, 70 EOC biopsies and 17 para-cancerous tissues with complete medical information were collected and analyzed. The expression of the androgen receptor (AR) was detected by immunohistochemistry. In addition, ovarian cancer cell lines were used for *in vitro* studies to further explore the role of androgen in cell proliferation and the possible mechanisms.

**Results:** The results showed that the expression of AR in ovarian cancer tissues was significantly elevated compared to the para-cancerous tissues, particularly in low-grade EOC, and the presence of high AR expression often suggested a worse prognosis. The *in vitro* study indicated that testosterone promoted the proliferation of the AR-positive SKOV3 cell line, which could be blocked by flutamide, but not in the AR-negative A2780 cell line. Next, we showed that testosterone-promoted proliferation in SKOV3 cells was abolished after we knocked out the AR. The mechanism studies revealed that the p-AKT expression in the ovarian cancer tissue was increased compared to the para-cancerous tissues, following a pattern similar to the increase of AR expression. Furthermore, the deletion and overexpression of SKOV3 cells' ARs lead to corresponding changes in the p-AKT levels. In addition, the BEZ235, an inhibitor of the PI3K/AKT signaling pathway blocked the proliferative effect of testosterone in SKOV3 cells.

**Conclusion:** We showed that testosterone was able to promote the proliferation of ovarian cancer cells through activating the PI3K/AKT signaling pathway in an AR dependent manner and AR may be a screening indicator for anti-androgen therapy sensitive cases of EOC.

## Introduction

Ovarian cancer is one the most fatal gynecological cancers and the fifth most common cause of cancer-related deaths in women worldwide 1, 2. It had caused an estimate of 184,799 deaths worldwide in 2018 3. EOC is the most common type among the different subsets of ovarian cancer, representing over 85% of all cases 4. Primary cytoreductive surgery followed by platinum-based chemotherapy treatment is well-accepted in clinical settings and has been the first-line therapy for many years. However, although many advances have been achieved in surgery and chemotherapy over the last 30 years, there has been no remarkable improvement in the five-year survival of patients with advanced EOC 5. For these reasons, a further understanding of the molecular pathogenesis leading to EOC and new therapeutic targeting is of critical necessary for improvement of the patient survival rate.

As we know, the ovary is not only an endocrine organ but also a target of and regulated by sex steroid hormones. Epidemiology studies have suggested that EOC usually occurs in postmenopausal women 6. The age when patients were first diagnosed ranged from 50 to 84 years old in Australia and Western countries and less than 65 years old in Asia and the Middle East 7. After menopause, the estrogen secretion abruptly declines, while androgen levels are gradually reduced and maintained for many years 8-11. Therefore, androgen is in a state of relative or absolute excess in postmenopausal women 12. Several studies have shown that androgen contributes to the pathogenesis of ovarian cancer 8, 13. For instance, a study 14 was conducted on androgen concentrations and EOC risk by detecting the pre-diagnosis androgen levels of 1,331 EOC cases and 3,017 control cases, and the results showed that testosterone was positively associated with EOC risk. The use of exogenous androgen has been proven to increase the risk of EOC 15-17 and oral contraceptive use, characterized by decreased androgen levels, reduces the risk of EOC 18, 19. However, in the phase II clinical study of antiandrogen therapy for EOC, neither flutamide nor bicalutamide showed good antitumor effects and did not improve patient survival 20-22.

Based on the above contradictions, on the one hand, it is necessary to find the criteria for screening sensitive cases; On the other hand, the mechanism of action of androgens needs further study. It is known that androgen can not only play a role through the genomic effect that bind to AR, but also through the non-genomic effect. The present study aimed to assess the specific role of AR in androgen carcinogenesis and whether it can be used as a screening indicator for patients sensitive to antiandrogen therapy and further explore the possible mechanism by which they contribute to the pathogenesis.

## Materials and Methods

The present study was approved by the Ethics Committee of the Second Hospital of Hebei Medical University. All subjects provided written informed consent.

### Human tissue specimens and TMA

Cancer biopsies were obtained from patients with EOC admitted to the Second Hospital of Hebei Medical University from 2010 to 2013. All patients were confirmed by pathology and had complete clinic data and were followed up to 5 years post-surgery. Exclusion criteria: patients receiving endocrine therapy within the last 1 year, including oral contraceptives; Patients with primary malignant tumor combined with other systems; Patients with severe systemic infection, hyperthyroidism, hypothyroidism and other endocrine diseases. All samples were embedded in paraffin and incorporated into tissue microarrays by Shanghai Outdo Biotech Co., Ltd. There were 87 tissue cores with a diameter of 1.5 mm in a microarray block. The microarray included tissues from patients with papillary serous carcinoma (PSC, N=48), endometrioid ovarian carcinoma (EMC, N=6), mucinous ovarian carcinoma (MUC, N=12), clear cell ovarian carcinoma (CCC, N=4) and adjacent normal tissues to tumor (para-cancerous tissues, Para-ca, N=17). All samples involved in the study were obtained from perimenopausal or postmenopausal women. The patients' characteristics are shown in Table 1.

### Immunohistochemistry (IHC)

We used immunostaining to detect the expression of androgen receptor (AR), AKT, and phosphorylated AKT (p-AKT). The IHC was performed using 5-mm sections of paraffin-embedded ovarian tissue. The tissue was deparaffinized in xylene, hydrated in a serial grading of alcohol solutions, incubated in an antigen recovery solution (sodium citrate buffer 0.01 Mol/L, pH 6.0) in a pressure cooker, and boiled for 5-6 min. Then, following the manufacturers' instructions of SP kit (ZSGB-BIO, Beijing, China) and DAB kit (ZSGB-BIO, Beijing, China), the samples were incubated overnight at 4°C with the primary antibody and rinsed with tris-buffered saline with Tween (TBST) three times before incubation with 2^nd^ antibody (antibodies used were: AR, 1:250, rabbit monoclonal, cat#:ab133273, Abcam; AKT, 1:300, rabbit monoclonal, cat#:4691, Cell Signaling Technology; p-AKT, 1:100, rabbit monoclonal, cat#:4060, Cell Signaling Technology). The negative controls were incubated without the primary antibody. After rinsing with TBST, the slides were examined by 2 independent pathologists. The expression levels of the target protein were scored based on the percentage of cells that stained positive (0: no positive cells; 1: ≤ 10% positive cells; 2: 11‒50% positive cells; 3: 51‒90% positive cells; and 4: ≥ 91% positive cells). Scores of 0 and 1 were considered low expression level, and scores of 2‒4 were considered high expression level. To better measure the expression of AR, TissueQuest software (TissueGnostics) was used, and results were given as the percentage of tissue stained positive per millimeter squared of total specimen area. The positive value was 1% or more.

### Cell culture and cell proliferation assay

The ovarian cancer cell lines SKOV3 and A2780 were purchased from the ATCC and cultured respectively in RPMI 1640 (Gibco) medium and Dulbecco's Modified Eagle Medium (DMEM, Gibco) supplemented with 10% fetal bovine serum (FBS, Gibco), 1 U/ml of penicillin (Gibco), and 1 mg/ml of streptomycin (Gibco). Cells were maintained at 37°C under a humidified atmosphere of 5% carbon dioxide. The cell passage was restricted within 30 generations. To measure the proliferation rate, the cells were seeded on a 96-well plate at 5.0 × 10^3^ cells per well and exposed to different conditions. At the indicated time points, the cell viability was determined by MTS assays (CellTiter 96® AQueous One Solution Cell Proliferation Assay**,** cat#: G3581, Promega, USA).

### Immunofluorescence

The cells were seeded on precoated wells of 12-well plates. The cells were first washed with 0.01 M of ice-cold phosphate-buffered saline (PBS) and fixed with 1 ml of ice-cold 4% paraformaldehyde for 10 min at room temperature (RT). Then, the cells were washed 3 times with PBS and blocked for 30 min with PBS-T (0.5% Triton X-100) containing 5% donkey serum at 37℃. After blocking, the cells were incubated with the primary antibody overnight at 4 °C (AR, 1:250, Rabbit monoclonal, cat#: ab133273, Abcam), followed by rinsing and incubation with the secondary antibodies (donkey anti-rabbit IgG (H+L) highly cross-adsorbed secondary antibody Alexa Fluor® 594, 1:1,000, cat#: A-21207, Thermo Scientific™) for 2 h in the dark at RT. The final step was to seal the slide onto the object slide with 4′,6-diamidino-2-phenylindole (DAPI) gel after washing with PBS. The images were than observed under an inverse Olympus FV1200 microscope.

### Western blot

The cells were harvested in radioimmunoprecipitation assay (RIPA) lysis buffer (Solaibio, cat#: R0020, China) supplemented with protease and phosphatase inhibitors (Protease Inhibitor PMSF, Solaibio, cat#: R0020, China and PhosSTOP, Roche, cat#: 04906845001, Switzerland). The protein concentration was measured using a BCA Protein Assay kit (Solarbio, cat#: PC0020, China). The protein samples were mixed with 4× sample buffer (Solarbio, cat#: P1015, China) and boiled for 5 min at 95°C for denaturation. Western blotting was performed according to standard protocols. The samples were separated by sodium dodecyl sulfate polyacrylamide gel electrophoresis (SDS-PAGE, 60 V at RT) and transferred onto polyvinylidene difluoride (PVDF) membranes (90 V at 4°, 90 min). Then, the membranes were blocked with 5% non-fat dry milk, dissolved in TBST buffer for 2 h, and incubated with the primary antibodies (AR, 1:2,000, rabbit monoclonal, cat#:ab133273, Abcam; AKT, 1:1,000, rabbit monoclonal, cat#:4691, Cell Signaling Technology; p-AKT, 1:2,000, rabbit monoclonal, cat#: 4060,Cell Signaling Technology; GAPDH, 1:1,000, rabbit polyclonal, cat#: AB_2619673, ABclone Technology) overnight at 4°C. Next, the membranes were rinsed with TBST buffer (3 times, 5 min each) and incubated with the secondary antibody ((rabbit IgG (H&L) Antibody Dylight™ 800 Conjugated, cat#: 610-445-002, dilution 1:10,000) at RT in the dark for 2 h. The membranes were rinsed again, and the results were analyzed by an Odyssey IR fluorescence scanning imaging system (LI-COR, USA). Both the incubation and washing processes were carried out on a shaker table.

### Transfection

The cells were transfected with plasmids containing the gene of interest (Sangon Biotech (Shanghai) Co., Ltd.) using Lipofectamine 2000 (Invitrogen, 11668-019) transfection reagent. The cells were seeded on 6-well plates in 2% FBS DMEM/1640 medium. Once the cells attached to the plate, they were transfected with the plasmids (final DNA used per well: 2,500 ng) using 10 µl of Lipofectamine 2000 per well following the manufacturer's protocols. The transfection reagents were removed after 12 h, and the cells were treated as indicated in each experiment. Western blotting and reverse transcription polymerase chain reaction (RT-PCR) were used to verify the transfection efficiency.

### RNA isolation and RT-PCR

The total RNA was isolated from the cells with TRIzol® reagent according to the manufacturer's instructions. The concentration and purity of the RNA were determined by spectrophotometry (A260:A280). The GoScript™ Reverse Transcription System (Promega, cat#: A5001, USA) was applied to synthesize cDNA according to the manufacturer's protocol. Briefly, the total RNA (2 μg) was used in reverse transcription reactions in a total volume of 20 μl with the following three-step incubation: 25°C for 5 min, 42°C for 60 min, and 70°C for 15 min. For amplification, the mixture, which comprised the cDNA sample (2 µl), gene-specific primers (2 µl), 2×Taq PCR MasterMix (TIANGEN, cat#: KT201, China; 12.5 µl) and ddH2O (8.5 µl), was incubated at 94°C for 3 min before being subjected to 40 cycles that consisted of 94°C for 30 s, 60°C for 30 s, and 72°C for30 s, followed by a final elongation phase at 72°C for 10 min. The RNA was visualized by agarose-formaldehyde gel electrophoresis, and Goldview™ (1:10000) was added to the gel before solidification. The sequences of the primers were as follows: AR forward, 5′ GGACGACCAGATGGCTGTCATTC 3′ and reverse, 5′ GCGAAGTAGAGCATCCTGGAGTTG 3′; GAPDH forward, 5′ TGACTTCAACAGCGACACCCA 3′ and reverse, 5′ CACCCTGTTGCTGTAGCCAAA 3′.

### Statistics

The data were processed with the software SPSS 23.0 and GraphPad Prism 7. The analysis of the numeric data relied on t-tests and non-parametric tests. A chi-squared test was applied to compare the categorical data. The overall survival (OS) was compared with the Kaplan-Meier method. The critical value was set as *p* < 0.05.

## Results

### Protein levels of AR in the TMA and its clinical relevance

**Figure 1:**
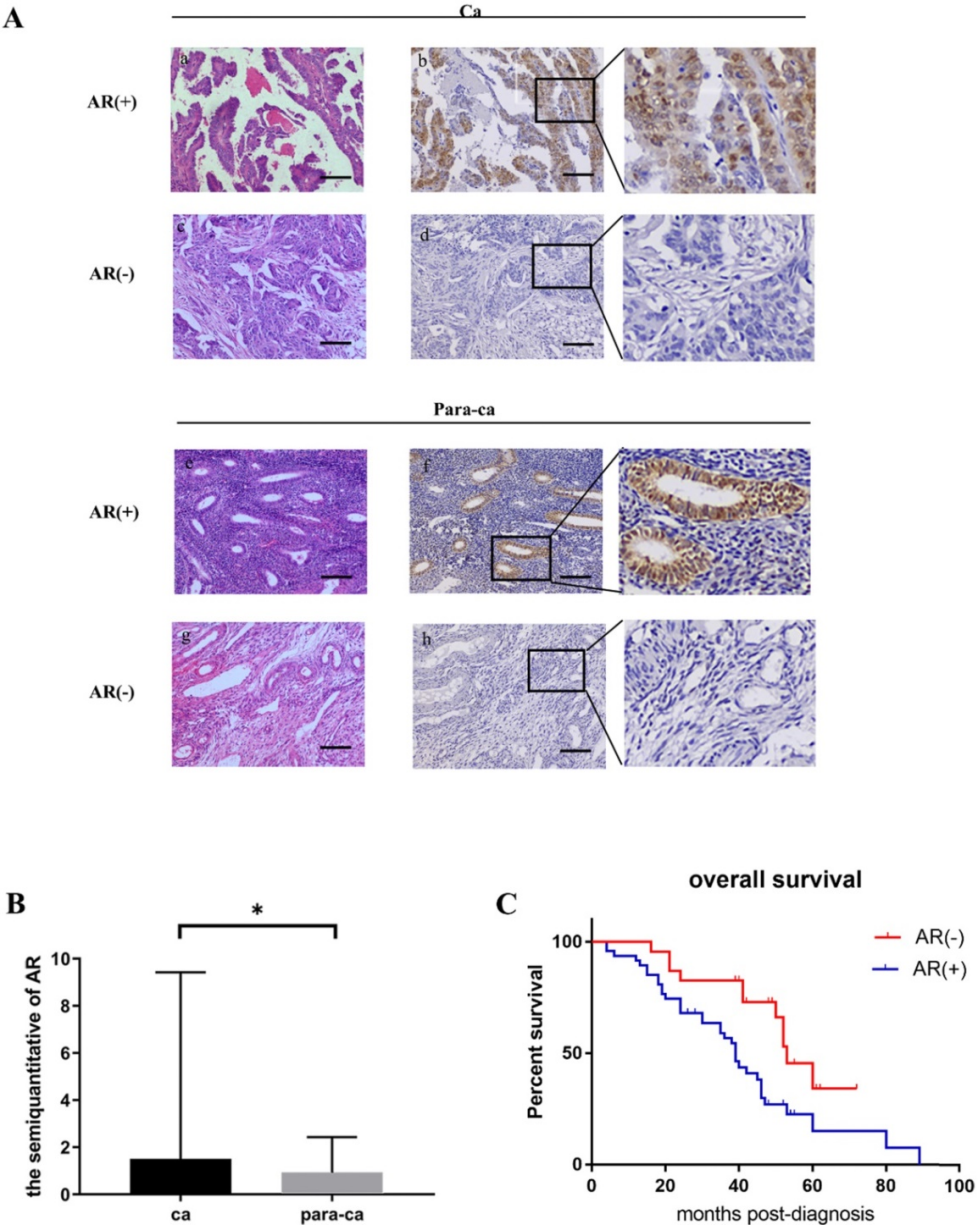
** Protein levels of AR in ovarian epithelial tissue and the clinical significance of AR. (A)** Tissue microarray immunohistochemical staining of AR: representative positive and negative IHC staining of AR (b, d) and corresponding HE staining in ovarian cancer tissues (a, c); Representative positive and negative IHC staining of AR (f, h) and corresponding HE staining in ovarian para-cancerous tissues (e, g). As a negative control, the primary antibody was omitted. Scale bar represents 100 μm. **(B)** Semiquantitative analysis of AR in ovarian cancer tissues and ovarian para-cancerous tissues. Immunostaining is expressed as the median with interquartile range. The analysis of the numeric data relied on non-parametric tests. *p<0.05; **p<0.01; ***p<0.001. **(C)** Kaplan-Meier plot analysis of EOC patients. Comparison of overall survival in AR positive vs. negative cases revealed that AR positivity is strongly associated with a shortened overall survival (*p*=0.009).

The IHC staining showed that the majority of AR was expressed in the nucleus (Fig. 1A). The semiquantitative analysis of the AR expression in the tissue microarray chips was performed by TissueQuest software (TissueGnostics) and the result suggested that the AR protein expression level in the ovarian cancer tissues was significantly higher compared to the para-cancerous tissues (Fig. 1B). We also found that the expression of the AR protein was associated with differentiated tumor grades. We showed that lower grade tumors was associated with increased AR expression (*P*=0.041). However, such correlation was not discovered regarding the different pathological subtypes (*P*=0.129). Furthermore, our data suggested that there was no association between the AR protein and age, the serum level of CA125, FIGO, or clinical stages (Table 2).

Moreover, the Kaplan-Meier method was applied to analyze whether the expression of AR affects the survival time of EOC patients. The results showed that the prognosis of the patients in the AR-positive group was worse compared to the AR-negative group (*P*=0.009) (Fig. 1C).

### Testosterone stimulated the growth of AR-positive ovarian cancer cells

**Figure 2:**
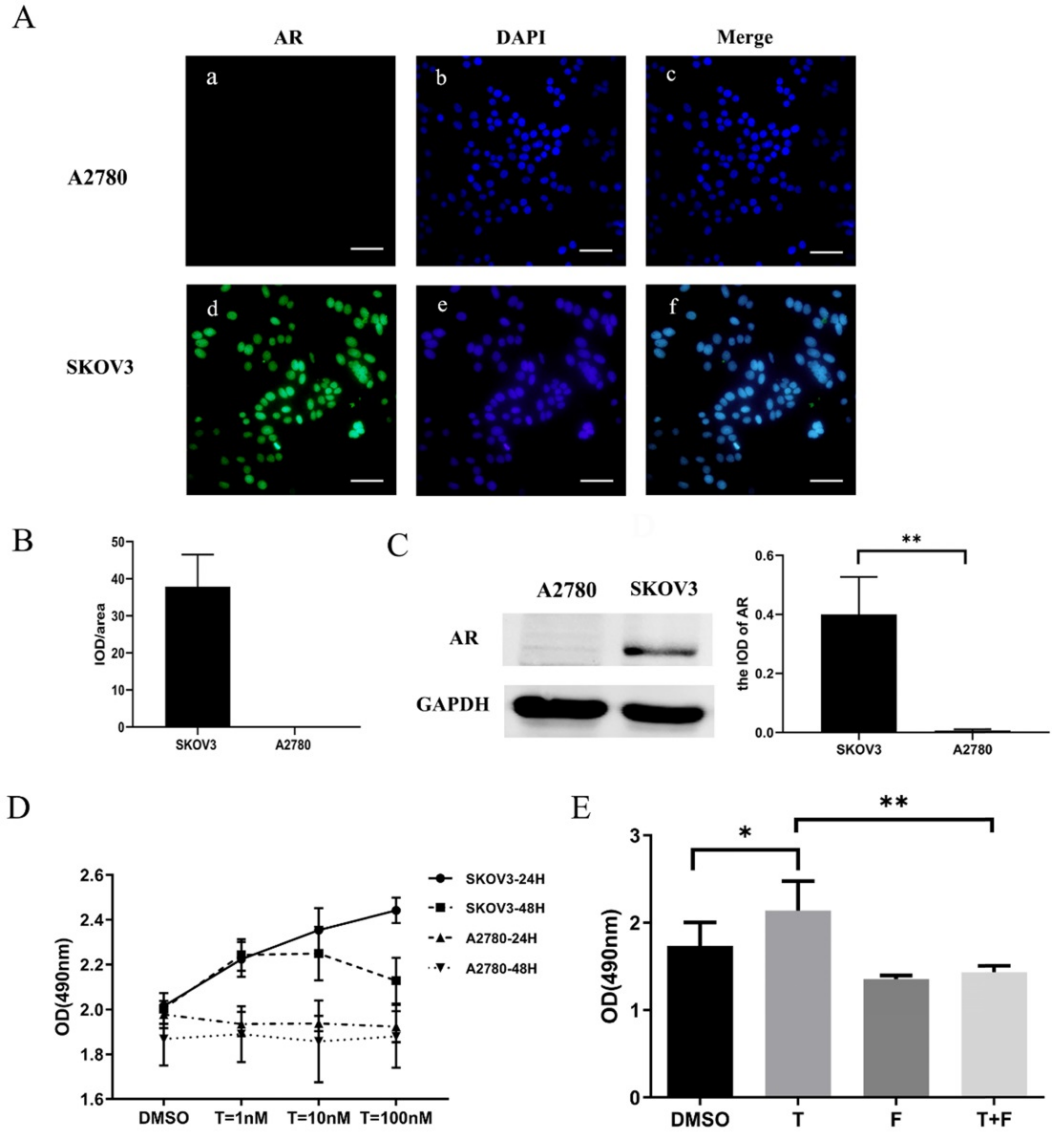
** Testosterone stimulated the growth of AR-positive ovarian cancer cells. (A)** Immunofluorescence detection of AR in ovarian cancer cell lines. Scale bar represents 50 μm. **(B)** Semiquantitative analysis of AR in the two ovarian cancer cells. **(C)** Western blotting analysis was performed to detect AR in SKOV3 and A2780 cell lines. Protein AR was detected as bands with a molecular mass of 100 kDa. **(D)** Analysis of the effects of different concentrations of testosterone on cell proliferation revealed that 10 nM and 100 nM testosterone significantly promote SKOV3 cell proliferation (*p*=0.000) in the 24h group, but there was no significant difference between the two concentrations (*p*=0.653). All different concentrations of testosterone had no effect on proliferation in the A2780 cell line (24h: ***p***=0.481; 48h: *p*=0.982). **(E)** Flutamide blocked the proliferative effects of testosterone on the SKOV3 cell line (*p*=0.01). *p<0.05; **p<0.01; ***p<0.001.

The expression of the AR protein in ovarian cancer cell lines was determined by cell immunofluorescence and western blot. Our data suggested that the SKOV3 cell was found to be AR-positive, while the A2780 cell was negative (Fig. 2 A, B, C).

To determine whether testosterone was associated with proliferation in ovarian cancer cells and to titrate an appropriate concentration, different amounts of testosterone (ranging from 1 nM to 100 nM) were added to SKOV3 and A2780 cells for 24/48 h before the proliferation rates were measured. The results showed that testosterone enhanced cell proliferation only in the SKOV3 cells but not the A2780 cells. After the 24-h treatment, the cell number increased by 10.45% (T=1 nM), 16.92% (T=10 nM), and 21.39% (T=100 nM) in the SKOV3 cells compared to controls, and all were found to be significant by statistical analysis (Figure 2D). It is important to point out that there were no differences between the T=10 nM and T=100 nM groups. However, when the testosterone treatment was prolonged to 48 h, the cell number increased by 12.15% (T=1 nM), 12.45% (T=10 nM), and 6.35% (T=100 nM), which were significantly lower than the 24-h treatment (Fig. 2D). In conclusion, when the concentration of testosterone was 10 nM and the time was 24 h, the effect on the proliferation of ovarian cancer cells was the most obvious.

### Testosterone-induced proliferation of SKOV3 cells was blocked by flutamide

We have shown that testosterone could stimulate proliferation only in the AR-positive SKOV3 cells but not the AR-negative A2780 cells. To determine whether the AR protein was indeed involved in the testosterone-induced proliferation, SKOV3 cells were pretreated with flutamide, a competitive antagonist of AR, for 1 h in indicated groups. An MTS assay was used to detect the proliferation rate of the SKOV3 cells. As shown in Fig. 2E, compared to the cells treated with testosterone alone, the enhanced proliferation was almost entirely abolished in the flutamide pre-treated cells (F+T group).

### Testosterone-induced proliferation of SKOV3 cells was mediated by AR

**Figure 3:**
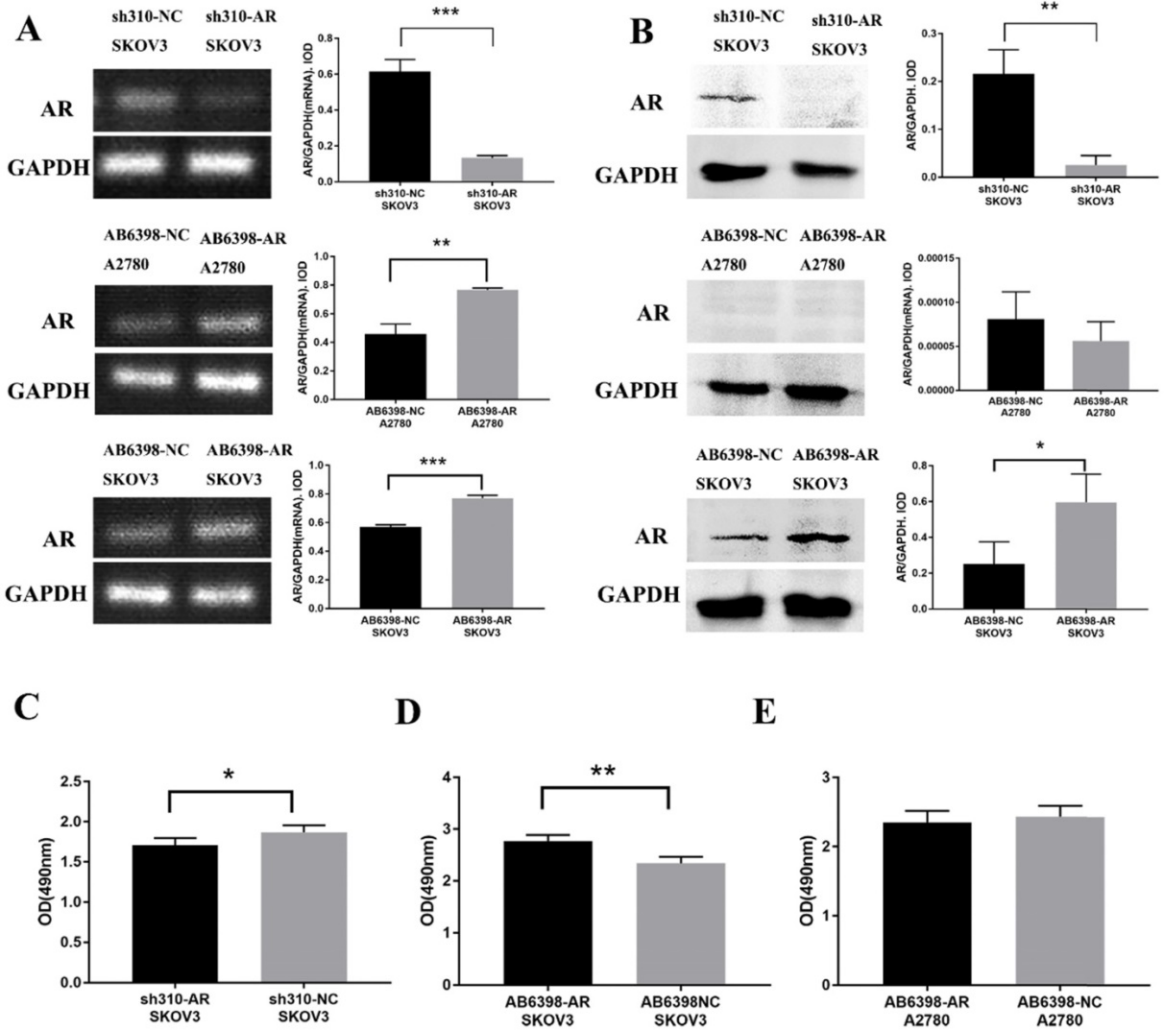
** Testosterone effects on proliferation in SKOV3 cells is mediated by AR. (A)** Changes in AR-mRNA after the plasmid knockout and overexpression of AR. **(B)** Changes in the AR protein after the plasmid knockout and overexpression of AR. **(C)** Knockout of AR reduced the proliferation effect of testosterone on SKOV3 cells (*p*=0.043). **(D)** Overexpression of AR increased the proliferation effect of testosterone on SKOV3 cells (*p*=0.001). **(E)** Overexpression of AR had no effect on the proliferation effect of testosterone on A2780 cells (*p*=0.456). *p<0.05; **p<0.01; ***p<0.001.

To further assess the importance of AR in testosterone-induced cell proliferation, sh310-AR (Sangon Biotech (Shanghai) Co., Ltd.) was used to knock down the AR expression in SKOV3 cells. In the meantime, AB6398-AR (Sangon Biotech (Shanghai) Co., Ltd.) was used to enhance the AR expression in SKOV3 cells and A2780 cells. The transfection efficiency was determined by RT-PCR (Fig. 3A) and western blot (Fig. 3B). The cells were then treated as indicated above, and the proliferation was measured by MTS assay. Our results indicated that the sh310-AR markedly reduced the AR mRNA and protein expression levels in SKOV3 cells, and the testosterone-enhanced proliferation were abolished (Fig. 3C). In contrast, the AB6398-AR increased the AR mRNA and protein expression levels and enhanced the testosterone-related proliferation (Fig. 3D). However, in A2780 cells, the AB6398-AR only upregulated the AR mRNA level but did not affect the protein expression; thus, no difference of proliferation was observed (Fig. 3 A, B and E).

### Testosterone and its receptor promoted growth of SKOV3 cells via the PI3K/AKT signaling pathway

**Figure 4:**
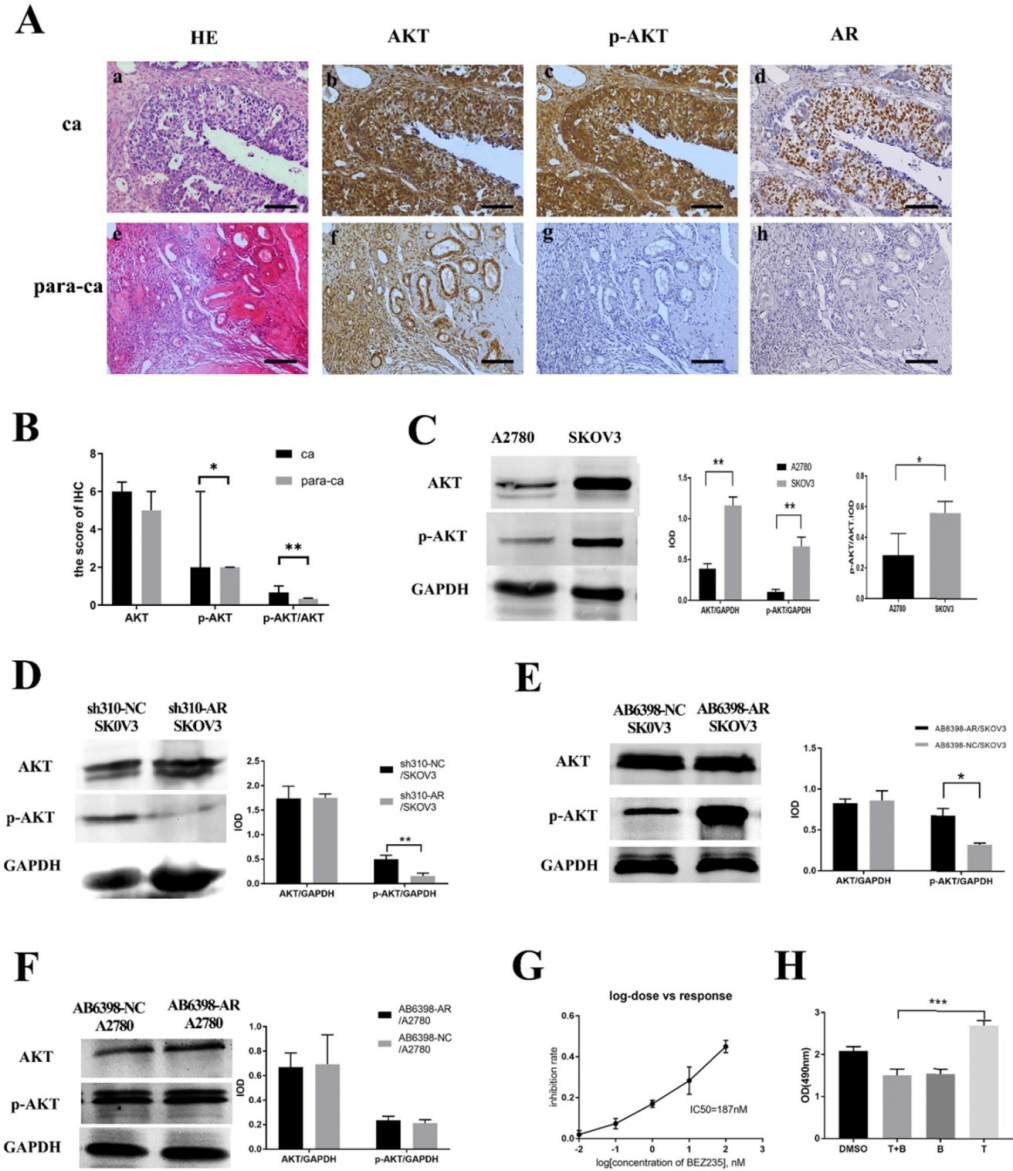
** Testosterone and AR promote growth in SKOV3 cells via the PI3K/AKT signalling pathway. (A)** Tissue microarray immunohistochemical staining of AKT, p-AKT, and AR and corresponding HE staining in ovarian cancer tissues and ovarian para-cancerous tissues. As a negative control, the primary antibody was omitted. Scale bar represents 100 μm. **(B)** Semiquantitative analysis of AKT, p-AKT and the value of p-AKT/ AKT. The scores of AKT and p-AKT are expressed as the median with interquartile range ( M(QR) ) and the analysis of the numeric data relied on non-parametric tests. The value of p-AKT/ AKT is expressed as mean with standard deviation(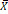
± S) and the analysis of the numeric data relied on t-test. **(C)** Western blotting analysis of AKT and p-AKT was performed in the SKOV3 and A2780 cell lines. Protein levels of AKT and p-AKT were detected as bands with a molecular mass of 60 kDa. **(D, E and F)** Expression of AKT and p-AKT in the cells after the knockout and overexpression of AR. **(G)** BEZ235 concentration vs inhibition rate curve (IC50=187 nM), and **(H)** BEZ235 blocked the proliferative effects of testosterone in the SKOV3 cell line (*p*<0.001).

Next, we investigated the potential mechanism of androgen/AR-induced proliferation observed in ovarian cancer cells. Immunohistochemistry (IHC) was used to detect the AKT and p-AKT expression in the tissues. Our results suggested that the p-AKT protein expression level in ovarian cancer tissues was remarkably higher compared to the para-cancerous tissues, while such difference was not observed in terms of the AKT expression (Fig. 4A, B). The p-AKT/AKT ratio in the ovarian cancer tissues was higher than that in the para-cancerous tissues (*P*=0.005). The western blot results indicated that both AKT and p-AKT protein levels in the SKOV3 cells were increased compared to the A2780 cells. Similarly, the p-AKT/AKT ratio in the SKOV3 cells was increased compared to the A2780 cells (Fig. 4C).To further confirm our hypothesis, the sh310-AR and AB6398-AR were transfected into the two cell lines and treated as indicated above. Our data showed that the p-AKT expression declined after the sh310-AR knocked down the AR expression in SKOV3 cells (Fig. 4D). Meanwhile, the p-AKT expression increased after the AB6398-AR upregulated the AR expression (Fig. 4E). However, in the A2780 cells that did not express AR protein, we observed no changes of AKT or p-AKT expression (Fig. 4F).

### BEZ235 inhibited the testosterone-induced growth in the SKOV3 cells

To further verify that testosterone and AR stimulated the growth of SKOV3 cells via the PI3K/AKT signaling pathway, BEZ235, a PI3K/AKT signaling inhibitor, was used to inhibit the signaling pathway. Firstly, we titrated the BEZ235 by treating the SKOV3 cells with different concentrations for 24 h, and an IC50=187 nM was obtained by SPSS analysis (Fig. 4G). Next, the BEZ235 and testosterone were used to treat the SKOV3 cells for 24 h as indicated. An MTS assay was used to measure the proliferation. The results showed that the testosterone-induced proliferation was completely abolished when co-culturing with the BEZ235 (Fig. 4H).

## Discussion

Androgen is an important hormone that regulates multiple physiology processes in women 23. Since it was first hypothesized to be involved in the pathogenesis of EOC relating to androgen in 1998, numerous studies have supported the idea that androgen can stimulate the proliferation of ovarian epithelium cells, thus contributing to the pathogenesis of EOC 24. In addition to classical genomic effects, androgens can also function through non-genomic effects that are independent of androgen receptors. In addition, androgens can be converted to estrogen in the female body. In order to explore the important role of AR in the process of androgen carcinogenesis, the TMA of human EOC tissues and adjacent normal tissues and EOC cell lines were involved.

Studies have shown that AR protein was stained positive in 90% 25 of EOC tissues by biochemical receptor assays and 43.7-84% by IHC 26, 27. In addition, numerous studies have consistently revealed that the AR is overexpressed in ovarian cancer cells compared to normal ovary cells. In the current study, we showed that the AR was stained positive in 67.1% of ovarian cancer tissues, which was significantly higher than that in para-cancerous tissues (29.4%).

**Figure 5:**
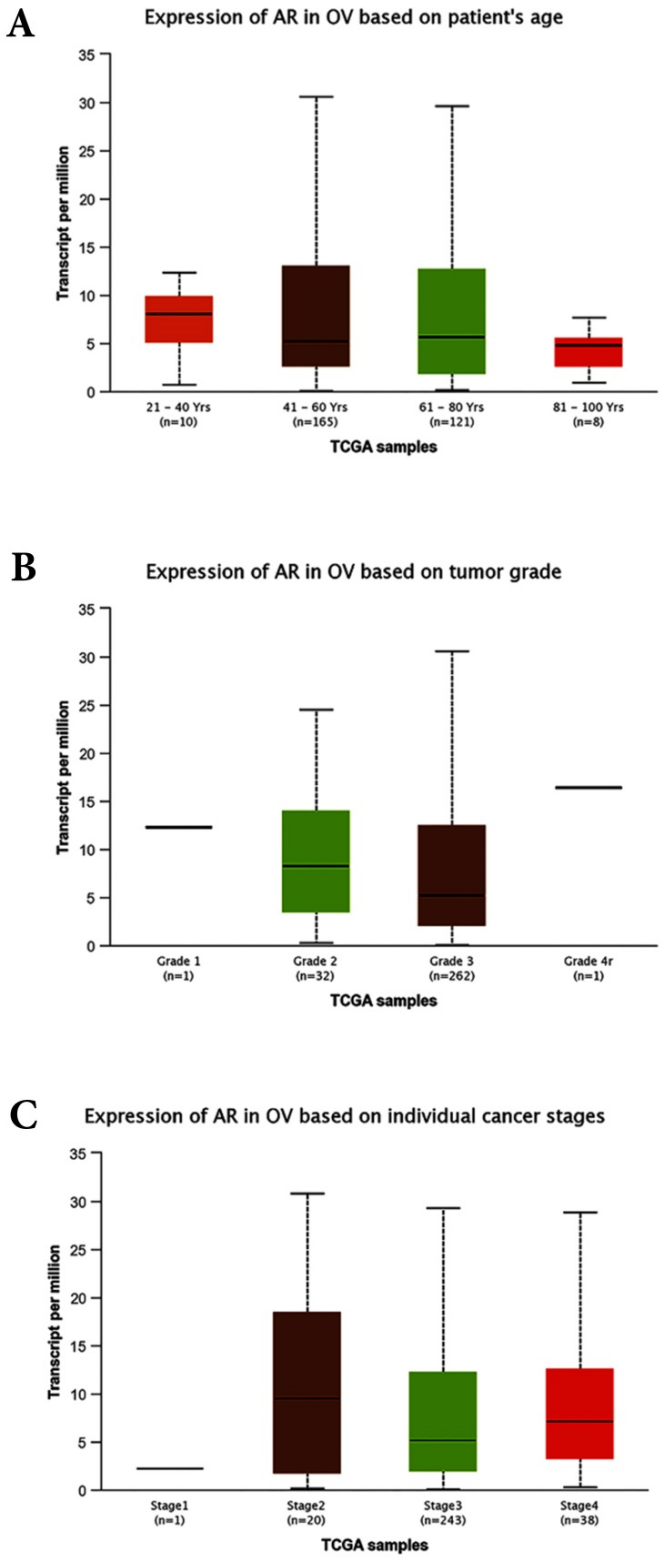
** Bioinformatics analyses based on the TCGA database. (A)** Expression of AR based on patient`s age (*p*>0.05). **(B)** Expression of AR based on tumor grades (*p*>0.05). **(C)** Expression of AR based on individual stages (*p*>0.05). OV: Ovarian serous cystadenocarcinoma.

The correlations between AR expression and disease characteristics, such as age, FIGO tumor stages, differentiated grades, and the serum level of CA125 were also evaluated in this study. The results revealed that the AR expression was not associated with patients' age, serum CA125 level, FIGO stages, clinical stages, or pathological subtypes. However, our data suggested that AR was highly expressed in tumors with lower grade. This finding seemed contradictory to some previous studies. A study involving 152 women with primary EOC demonstrated that the expression of AR was higher in more advanced stages but was not associated with tumor grades 28. In the preset study the cancer-related database TCGA (http://cancergenome.nih.gov) and the online tools for the analysis of data provided by TCGA (http://ualcan.path.uab.edu/index.html) were used to predict the expression levels of AR in the EOC. All of the cases were ovarian serous cystadenocarcinoma and unevenly distributed of race (Caucasian: 252, African-American:26, Asian: 12, and no Han Chinese). The bioinformatics analyses showed that the expression of AR was not associated with patients` age, tumor FIGO stages and tumor grades (Fig. 5A, B and C). Further research with a larger sample size is needed to further examine the association between AR expression and tumor grade/subsets.

The studies of AR expression and prognosis of EOC are inconsistent and we discovered that the prognosis of AR-positive patients was worse than that of AR-negative patients.

To further investigate the role of androgen receptor in the pathogenic process, EOC cell lines SKOV3 and A2780 were used in this study. The AR expression in these two cell lines were detected by immunofluorescence and western blot. The results suggested that the SKOV3 cells were AR-positive, while the A2780 cells did not express AR.

In this study, we observed that testosterone could only stimulate cell proliferation in the AR-positive SKOV3 cells and could be inhibited by flutamide. Next, the AR-dependent testosterone-induced proliferation was further confirmed in transfection assays where the testosterone induced proliferation rate changed consistently with the AR expression levels in SKOV3 cells. Our study suggested that androgen played an important role in cell proliferation in ovarian cancer cells in an AR-dependent manner. This provides evidence for screening sensitive cases with anti-androgen therapy by the expression of AR.

It is well accepted that the PI3K/AKT signaling pathway acts as a pivotal pathway and participates in various cellular activities under physiology conditions 4, 29, 30. However, it is also considered one of the most important tumor-related signaling pathways 31. The aberrant activation of this pathway has been proven to be closely related to tumorigenesis across a variety of malignancies 32.

The role of testosterone/AR in prostate cancer and breast cancer has been well studied. Testosterone is known to participate in the tumorigenesis via the PI3K/AKT signaling pathway. However, how testosterone contributes to the pathogenesis of EOC has not been well studied, and this was our next focus area of study. Our data discovered a significant increase in the expression of phosphorylated AKT (p-AKT), the active form of AKT, in the ovarian cancer tissues compared to the para-cancerous tissues, which is consistent with the AR expression, while there was no difference in the AKT expression. In addition, we showed the AKT and p-AKT levels in the AR-positive SKOV3 cells were significantly higher than those in the AR-negative A2780 cells. Similarly, the p-AKT/AKT ratio in SKOV3 cells was higher than that in the A2780 cell line. Furthermore, we modified AR protein expression by transfection assays in the SKOV3 cells and discovered that the p-AKT expression levels also changed accordingly, while the AKT level maintained the same. Next, we demonstrated that the effect of testosterone-induced SKOV3 cell proliferation could be inhibited by BEZ235, an inhibitor of the PI3K/AKT signaling pathway. Based on the above finding, we can safely draw the conclusion that the androgen/AR promoted the growth of ovarian cancer cells via the PI3K/AKT signaling pathway. However, a recent study suggested that the inhibition of PI3K resulted in an increased AR protein level on EOC cells 33. This may be due to these two oncogenic pathways cross-regulating each other by reciprocal feedback just like in prostate cancer 34. Moreover, the effects of androgen in early stages of carcinogenesis may differ from that in progressed stages. Therefore, more studies are needed to further explore the role of AR and the PI3K/AKT signaling pathway in the pathogenesis of EOC so that new therapeutic targets can be provided.

## Conclusion

In summary, our results suggested that the AR expression was significantly increased in ovarian cancer tissues, particularly in low-grade EOC, and the presence of high AR expression often suggested a worse prognosis. Next, we showed that testosterone was able to promote the proliferation of ovarian cancer cells through activating the PI3K/AKT signaling pathway in an AR-dependent manner. Overall, the results of the study identified androgen/AR as a candidate oncogene in EOC and a potential target for therapy and AR may be a screening indicator for antiandrogen therapy sensitive cases of EOC.

## Figures and Tables

**Table 1:** Patient characteristics (N = 87)

Characteristic	Group	
	Cancer (N = 70)	Para-cancer (N = 17)
**Age (years)** 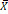 ±***s***	51.6±13.9	48.9±18.8
<55 n(%)	36(78.3)	10(21.7)
≥55 n(%)	34(82.9)	7(17.1)
**Gravidity** median(range)	3(2)	3(3)
**Parity** median(range)	2(2)	2(3.5)
**Histological subtype**		
Serous n(%)	48(68.6)	11(64.7)
Mucinous n(%)	12(17.1)	4(23.5)
Endometrioid n(%)	6(8.6)	2(11.8)
Clear cell n(%)	4(5.7)	0(0)
**FIGO stage**		
I-IIA n(%)	18(25.7)	5(29.4)
IIB-IV n(%)	52(74.3)	12(70.6)
**Grading**		-
1 -2n(%)	50(71.4)	6(35.3)
3-4 n(%)	18(25.7)	3(17.6)
Unknown n(%)	2(2.9)	8(47.1)
**Residual disease**		-
None n(%)	62(88.6)	12(70.6)
Present n(%)	8(11.4)	5(29.4)
**Chemotherapy**		-
Platinum based n(%)	60(85.7)	15(88.2)
None n(%)	10(14.3)	2(11.8)

**Table 2:** Clinical significance of AR in EOC

Characteristic	Cancer Group		
	AR (+) (N = 47)	AR (-) (N = 23)	*P*
**Age** (years) median(range)	54(24)	54(13)	0.689
**Gravidity** median(range)	3(2)	3(3)	0.730
**Parity** median(range)	2(2)	2(1)	0.547
**Histological subtype**			
Serous n(%)	35(72.9)	13(27.1)	0.129
Non-Serous n(%)	12(54.5)	10(45.5)	
**FIGO stage**			
I-IIA n(%)	15(68.2)	7(31.8)	0.900
IIB-IV n(%)	32(66.7)	16(33.3)	
**Tumour grade**			
1-2 n(%)	38(76)	12(24)	**0.041**
3-4 n(%)	9(50)	9(50)	
Unknown n(%)	0	2	
**CA125 U/ml** median(range)	600(921)	338.5(559.2)	0.063
